# Comparison of ocular morphological measurement by wide-angle echography and magnetic resonance imaging

**DOI:** 10.20407/fmj.2021-028

**Published:** 2022-05-25

**Authors:** Kohsuke Sekido, Kazuhiro Murayama, Tadashi Mizuguchi, Ryota Sakurai, Akiyoshi Iwase, Yoshiaki Shimada, Keita Suzuki, Atsuhiro Tanikawa, Masayuki Horiguchi

**Affiliations:** 1 Department of Ophthalmology, Fujita Health University, School of Medicine, Toyoake, Aichi, Japan; 2 Department of Radiology, Fujita Health University, School of Medicine, Toyoake, Aichi, Japan; 3 Department of Ophthalmology, Fujita Health University Bantane Hospital, Nagoya, Aichi, Japan

**Keywords:** Wide-angle ultrasound diagnostic device, Wide-angle B-mode echography, Magnetic resonance imaging

## Abstract

**Objectives:**

To compare the eye axial length (AL), equatorial horizontal diameter (HD), and equatorial vertical diameter (VD) of normal eyes using a novel wide-angle, arc-scanning, ultrasound diagnostic device for wide-angle B-mode echography.

**Methods:**

In this cross-sectional study, wide-angle B-mode echography and magnetic resonance imaging (MRI) were conducted on 22 normal eyes; the AL, HD, and VD were measured.

**Results:**

The mean ALs were as follows: wide-angle B-mode echography, 25.22±1.47 mm and MRI, 25.24±1.46 mm; a significant correlation was observed between the two measurements (β=0.995 [0.976, 1.013]; *p*<0.001; 95% R^2^=1.00). The mean HDs were as follows: wide-angle B-mode echography, 22.33±0.84 mm and MRI, 22.55±0.90 mm; a significant correlation was observed between the two measurements (β=0.902 [0.750, 1.179]; *p*<0.001; 95% R^2^=0.81). The mean VDs were as follows: wide-angle B-mode echography, 22.77±0.91 mm; and MRI, 22.88±0.92 mm; a significant correlation was observed between the two measurements (β=0.966 [0.853, 1.097]; *p*<0.001; 95% R^2^=0.93).

**Conclusions:**

There were no significant differences in the measurements for each parameter by wide-angle B-mode echography and MRI. Therefore, wide-angle B-mode echography permits accurate visualization of ocular morphology.

## Introduction

Ocular tomography using a B-mode ultrasound diagnostic device has been used in ophthalmology for many years; however, it can only examine up to a range of 52° in the central region of the eye. Therefore, we have developed a rotating ultrasound probe and a wide-angle B-mode echography device that enable whole-eye tomography. This device allows diagnostic imaging for peripheral lesions, at 100° for diseased eyes where the ocular fundus is not visible; it also allows assessment of whole-eye morphology.

In the current sector-scan method, the ultrasound transmission device is placed in a fixed position, and the sclera interferes with ultrasound transmission in images of the peripheral region; therefore, images of the periphery cannot be obtained. In attempts to reveal the relationships between ophthalmic lesions and ocular morphology, the axial length (AL) and horizontal and vertical diameters (HD and VD, respectively) have been measured using magnetic resonance imaging (MRI).^1–4^ Watanabe *et al.* and Atchison *et al.* reported the use of MRI to measure the eye axes, HD, and VD of normal and myopic eyes; they classified the ocular morphologies of myopic eyes into four types.^1,3^ Tanaka *et al.* have reported disease-related differences in ocular and vitreous humor volumes; they also demonstrated the clinical importance of imaging-mediated evaluation of whole-eye morphology.^5^ However, there are several financial and time-related restrictions for the measurement of ocular morphology by MRI; the use of MRI for diseases that require urgent treatment, even for research purposes (e.g., retinal detachment and crystalline lens dislocation), may contribute to further complications. Wide-angle B-mode echography, which allows imaging of whole-eye morphology, can be performed quickly and at a much lower financial cost. Sekido *et al.* have also demonstrated, using a wide-angle B-mode echography device, that the ocular HD and VD are greater in myopic eyes than in normal eyes.^6^ Similarly, positive correlations have been reported between AL, HD, and VD in normal eyes and increases in both HD and VD as the eye axis moves forward^6^; however, the accuracy of these findings remains unclear.

In the present study, MRI and wide-angle B-mode echography results of healthy eyes were used to ascertain the feasibility of wide-angle B-mode echography; ALs and equatorial diameters were compared.

## Methods

### Participant selection

The study included 22 healthy volunteers, comprising eight men and 14 women, who were recruited at Fujita Health University Hospital. In total, 22 eyes were examined, comprising 16 left and six right eyes. The mean age (±standard deviation) of the participants was 29.77±6.27 years (range, 22–50 years). All participants were of Asian ethnicity. Participants were excluded if they had ophthalmic diseases (e.g., posterior staphyloma or cataracts) or if the MRI could not be obtained for reasons such as claustrophobia or the presence of metallic implants in the body.

Before the examination, each participant received a detailed explanation of the study; they then provided written informed consent. This study protocol was approved by the Institutional Review Board of Fujita Health University (HM20-391) and was conducted in compliance with the principles outlined in the Declaration of Helsinki.

### Wide-angle ultrasound diagnostic device (wide-angle B-mode echography device)

**Figure 1:**
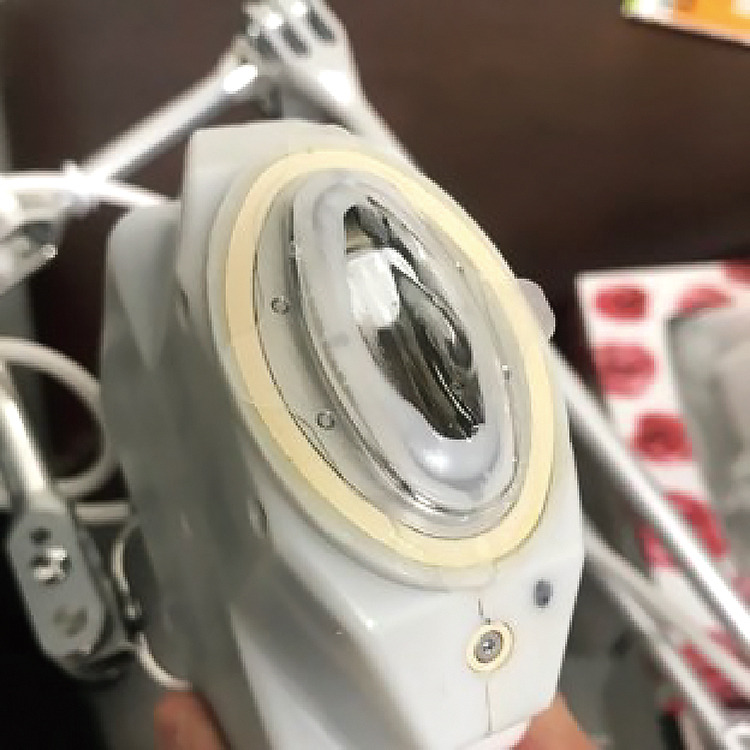
Probe photographs obtained using a wide-angle ultrasound diagnostic device A cap filled with a physiological saline solution was fitted and the tip of the contained probe was free to move to trace an arc (Extracted from Figure 1 from Sekido K, Murayama K, Mizuguchi T, Sakurai R, Tanikawa A, Horiguchi M. Eyeball in the normal eye evaluated using a wide-angle ultrasound diagnostic device. Folia Japonica de Ophthalmologica Clinica. 2020;13:717–21 with permission)

**Figure 2:**
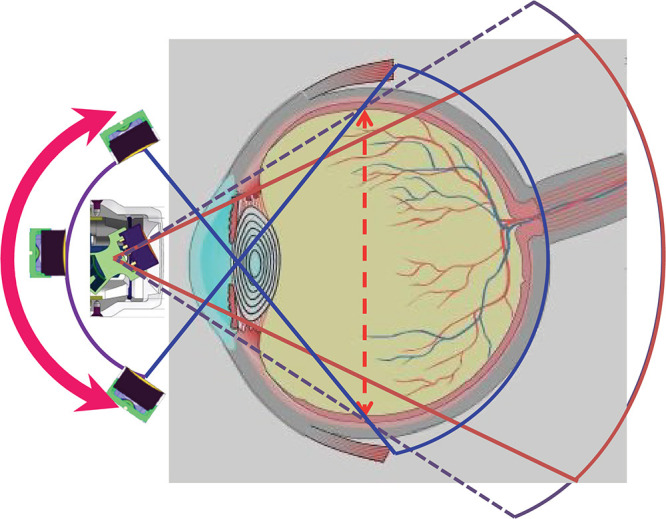
Arc-scan image obtained using a wide-angle ultrasound diagnostic device The wide-angle ultrasound diagnostic device can obtain images over a wider range, indicated by blue lines, compared with the conventional ultrasound diagnostic B-mode device, indicated by red lines

**Figure 3:**
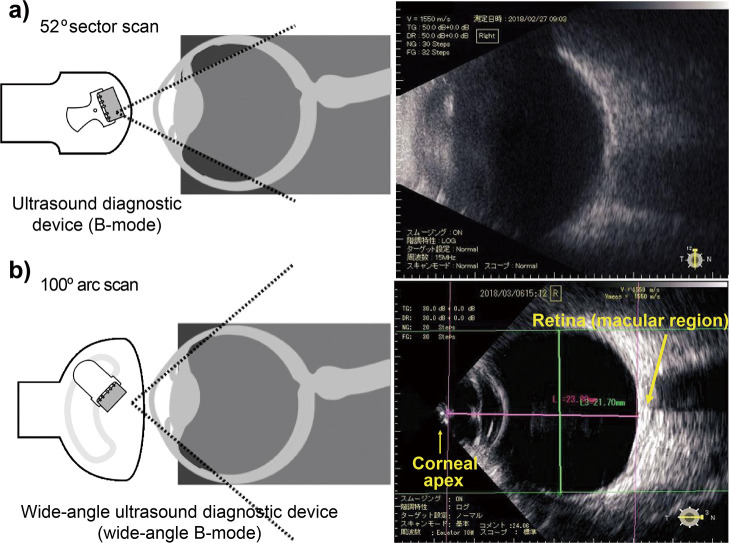
Images obtained using (a) a conventional ultrasound diagnostic B-mode device and (b) a wide-angle ultrasound diagnostic B-mode device The imaging range is wider with the wide-angle ultrasound diagnostic B-mode device than with the conventional B-mode device. The distance from the corneal apex to the retinal macular region, indicated by an arrow, was measured as AL; the maximum diameters across the peripheral retina of horizontal and vertical lines that intersected with the AL were defined as the HD and VD, respectively. AL, axial length; HD, horizontal distance; VD, vertical distance (Extracted from Figure 2 from Atchison DA, Jones CE, Schmid KL, Pritchard N, Pope JM, Strugnell WE, et al. Eye shape in emmetropia and myopia. Invest Ophthalmol Vis Sci. 2004;45:3380–6 with permission).

This device obtains wide-angle images by capturing the ultrasonic waves transmitted in various directions through the use of an arc scan (Figure 1) that enables the ultrasound transmission device to move in different directions (Figure 2). Figure 3 shows images obtained using B-mode devices and wide-angle B-mode echography devices during standard ophthalmological assessments.

After mydriasis of the eye had been achieved using tropicamide phenylene hydrochloride, 0.4% lidocaine hydrochloride eye drops were administered and hydroxyethylcellulose+boric acid+inorganic salt compound topical solution was applied to the cornea. Then, with the participant in the supine position, the eye was examined directly. A fixation point was placed on the ceiling, 2 m above the supine participant; the participant was instructed to focus the unexamined eye on the fixation point. An examiner confirmed the visibility of the optic nerve and the equator of the eyeball while the participant was staring straight at the fixation point; the examiner measured the AL, HD, and VD of the eyeball.

The AL was measured as the distance from the corneal apex to the retinal macular region. The maximum diameter across the peripheral retina of a horizontal line that intersected the AL was regarded as the HD, while the equivalent vertical line was regarded as the VD. Wide-angle ultrasound examinations on all participants were performed by certified orthoptists.

A-mode ultrasound

The AL was also measured by conventional A-mode ultrasound system (OA-2000: Tomey Corporation, Tokyo, Japan) that used the laser interferometry to measure the axial length in a non-contact manner.

### MRI

All MRI examinations were performed via the fast-field echo method with T1-weighted images using a 1.5T magnetic resonance system (Achieva 1.5T Nova Dual; Philips Healthcare, Best, the Netherlands) and a 47-mm microscopy coil. The pulse sequence parameters were as follows: repetition time, 9.9 ms; echo time, 4.4 ms; imaging angle, 25°; imaging matrix, 224×192; reconstruction matrix, 288×288; acquisition slice thickness, 1 mm; reconstruction slice thickness, 0.5 mm; field of view, 80×80 mm; and number of excitations, 2.

After mydriasis of the eye had been achieved using tropicamide phenylene hydrochloride, the examination was conducted with the participant in supine position. To suppress ocular movement, a 45° mirror was fixed 15 cm in front of the eye, vertically above the participant; the participant was instructed to stare at the fixation point. In addition, the participant was instructed to avoid blinking during imaging. After confirmation by both the ophthalmologist and radiology technician that the participant’s blinking was suppressed and their eye was focused on the fixation point, a sequence of images was obtained. If this confirmation was not possible, the position of the mirror was changed, and imaging was repeated.

### MRI data measurement methods

**Figure 4:**
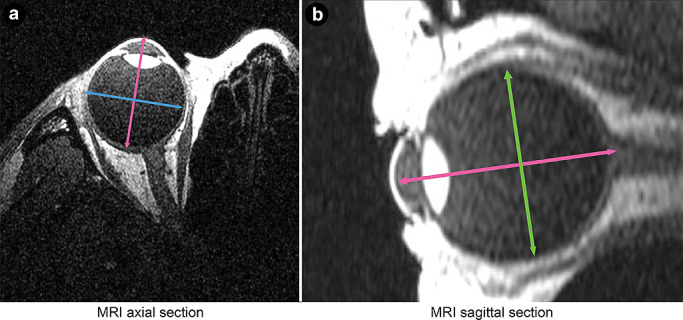
MRI images of (a) an axial section and (b) a sagittal section The AL, shown by red lines in both the axial and sagittal planes, is the distance from the corneal anterior surface to the approximate position of the fovea centralis. The HD, shown by the green line, is the maximum diameter across the retina of a horizontal line that intersects the eye axis on the axial plane image. VD is the maximum diameter across the retina of a vertical line that intersects the eye axis on the sagittal plane image. MRI, magnetic resonance imaging; AL, axial length; HD, horizontal distance; VD, vertical distance

**Figure 5:**
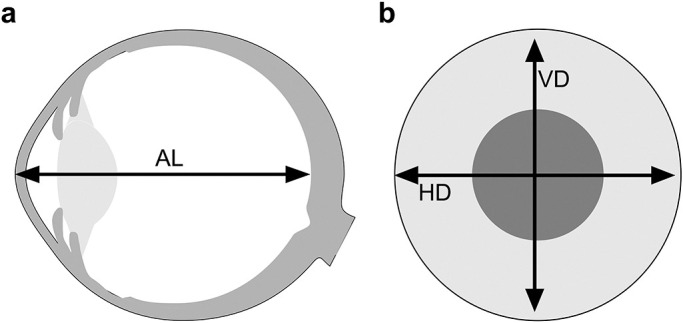
Schematic diagrams for each parameter (a) The distance from the corneal apex to the macular region is defined as AL. (b) The maximum diameter in the equatorial region of the peripheral retina is defined as HD. (c) The vertical diameter in the equatorial region that intersects the HD is defined as VD. AL, axial length; HD, horizontal distance; VD, vertical distance (Extracted from Figure 3 from Sekido K, Murayama K, Mizuguchi T, Sakurai R, Tanikawa A, Horiguchi M. Eyeball in the normal eye evaluated using a wide-angle ultrasound diagnostic device. Folia Japonica de Ophthalmologica Clinica. 2020;13:717–21 with permission).

Ocular measurements were performed using a scanning and image-processing console (Extend Workspace; Royal Philips, Amsterdam, the Netherlands). Central images were selected on the axial and sagittal planes; the AL was regarded as the distance from the corneal anterior surface to the approximate position of the retinal fovea centralis, in both the axial and sagittal planes. The HD in the equatorial region was the maximum diameter across the retina of a horizontal line that intersected the eye axis on an image obtained in the axial plane. The VD in the equatorial region was the maximum diameter across the retina of a line that vertically intersected the eye axis on an image obtained in the sagittal plane. Measurements were obtained to two decimal points; each of the parameters (AL, HD, and VD) was measured three times and mean values were calculated. The ocular morphological parameters were measured in millimeters; they are expressed as means with standard deviations. The MRI images are presented in Figure 4, while Figure 5 shows the schematic for each of the parameters in wide-angle B-mode echography and MRI.

### Statistical analysis

All statistical procedures were performed using SPSS^®^ Statistics Version 24 (IBM Corp., Armonk, NY, USA). A linear regression model was used to evaluate the correlations of each of the parameters (AL, HD, and VD) between measurements by both wide-angle B-mode echography and MRI. The lower and upper limits of the 95% confidence interval for the coefficient were used to estimate the accuracy of the linear regression coefficient. The 95% confidence interval (CI) of the mean was calculated for each parameter; the mean differences between wide-angle B-mode echography and MRI were also calculated. Pearson’s correlation coefficient and R^2^ values were obtained from the regression model. The significance level was set at two-tailed *p*<0.05.

## Results

### Axial length (AL)

**Figure 6:**
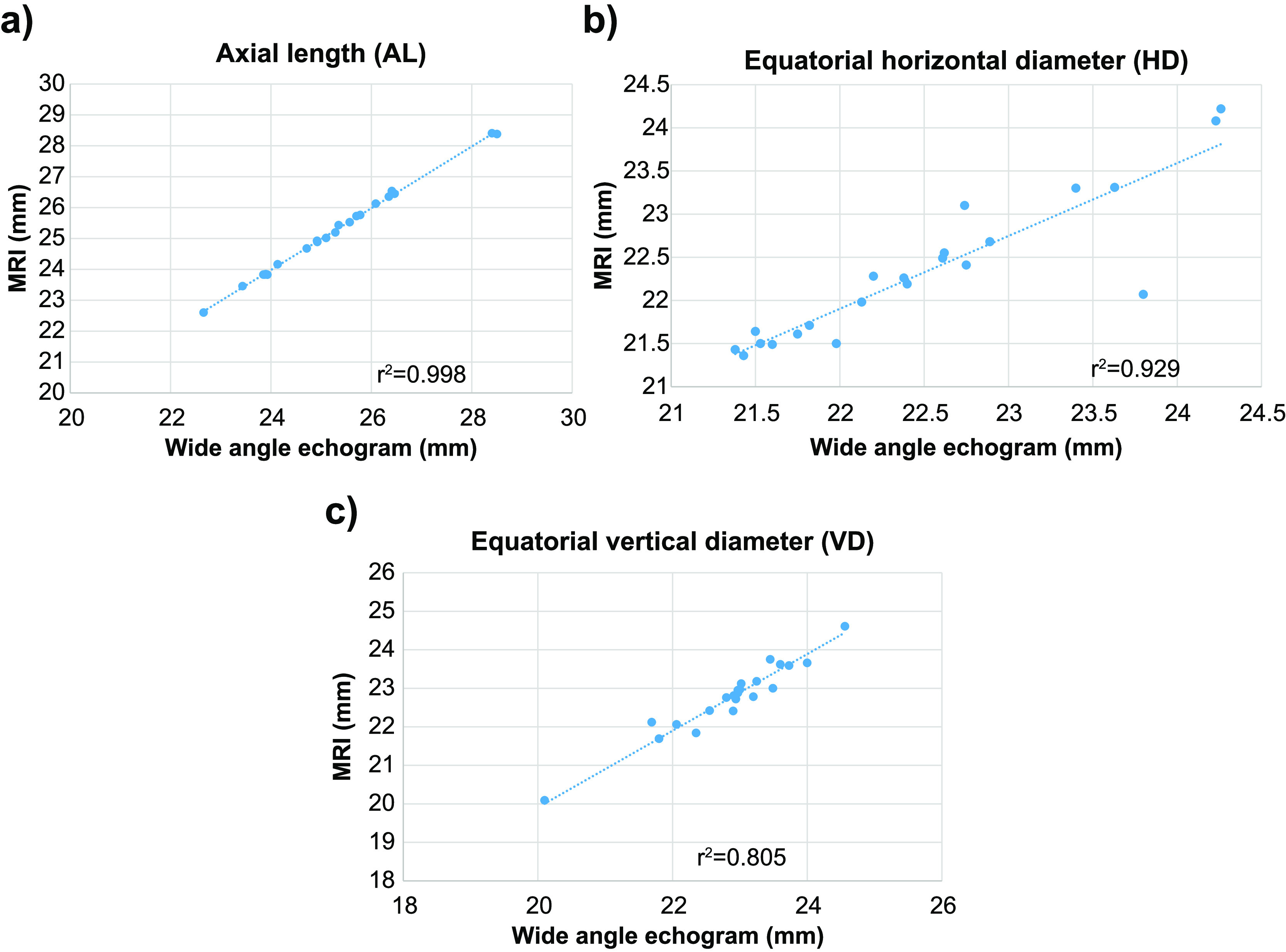
Scatter graphs and fitted curves for (a) AL, (b) HD, and (c) VD, obtained via wide-angle ultrasound diagnostic echography and MRI AL, HD, and VD demonstrated significant correlations between measurements made using wide-angle ultrasound diagnostic echography and measurements made using MRI. AL, axial length; HD, horizontal distance; VD, vertical distance; MRI, magnetic resonance imaging

The mean ALs of the 22 eyes, obtained by each method, were as follows: conventional A-mode ultrasound system (OA-2000: Tomey Corporation, Tokyo, Japan), 25.23±1.48 mm; wide-angle B-mode echography, 25.22±1.47 mm; and MRI, 25.24±1.46 mm. Significant correlations were observed between measurements performed using A-mode ultrasound and wide-angle B-mode echography (β=1.00 [0.996, 1.004]; *p*<0.001; 95% CI=–0.01 to 0.03), A-mode ultrasound and MRI (β=1.00 [0.995, 1.004]; *p*<0.001; 95% CI=–0.03 to 0.01), and wide-angle B-mode echography and MRI (β=0.995 [0.976, 1.013]; *p*<0.001; 95% CI=–0.32 to 0.01) (Table 1, Figure 6).

### Horizontal distance (HD)

The mean HDs of the 22 eyes, obtained by each method, were as follows: wide-angle B-mode echography, 22.33±0.84 mm and MRI, 22.55±0.90 mm. A significant correlation was observed between the two measurements (β=0.902 [0.750, 1.179]; *p*<0.001; 95% CI=–0.32 to 0.1) (Table 1, Figure 6).

### Vertical distance (VD)

The mean VDs of the 22 eyes, obtained by each method, were as follows: wide-angle B-mode, 22.77±0.91 mm and MRI, 22.88±0.92 mm. A significant correlation was observed between the two measurements (β=0.966 [0.853, 1.097]; *p*<0.001; 95% CI=0.00 to 0.21) (Table 1, Figure 6).

### Comparisons between each of the parameters

The HD/AL (HD to AL ratio) was as follows: wide-angle B-mode echography, 0.89±0.03 and MRI, 0.89±0.03. A significant correlation was observed between the two ratios (β=0.899 [0.642, 1.020]; *p*<0.001; 95% CI=0.00 to 0.01). The VD/AL (VD to AL ratio) was as follows: wide-angle B-mode echography, 0.91±0.05 and MRI, 0.91±0.05. A significant correlation was observed between the two ratios (β=0.975 [0.867, 1.070]; *p*<0.001; 95% CI=0.00 to 0.01). The HD/VD (HD to VD ratio) was as follows: wide-angle B-mode echography, 0.98±0.04 and MRI, 0.98±0.04. A significant correlation was observed between the two ratios (β=0.917 [0.763, 1.152]; *p*<0.001; 95% CI=0.00 to 0.01) (Table 1).

## Discussion

Multiple studies have been performed regarding the shape of the eyeball; they have mainly used either the A-scan method (conventional B-mode ultrasound diagnostic device)^7–13^ or MRI.^1–4,13–15^ However, measurement of the equatorial diameters of the eyeball using a conventional B-mode ultrasound diagnostic device is challenging; to our knowledge, no studies have accurately measured equatorial and axial diameters using B-mode ultrasonography.

In the present study, HD and VD were measured using wide-angle B-mode echography; the results were compared with findings obtained by MRI. For each participant, these two sets of measurements were performed on the same eye, with the participant in the same body position, with the establishment of mydriasis confirmed, and—as much as possible—with similar imaging conditions. AL, HD, and VD measured by wide-angle B-mode echography and MRI showed strong correlations (Table 1). In addition, analysis of the measurements for each parameter by wide-angle B-mode echography, including consideration of the 95% CI, showed no significant differences from the measurements obtained by MRI; hence, wide-angle B-mode echography appears to enable accurate determination of ocular morphology.

Furthermore, our novel wide-angle B-mode echography device increases the ease, reduces the cost, and decreases the time of eye morphology measurement compared with MRI, while maintaining an accuracy similar to the accuracy of MRI.

This study had some limitations. The participants in this study were young, healthy individuals with good binocular vision; hence, there were no participants who performed activities of daily living poorly, had difficulty during the examination, or had low vision in both eyes. To more fully investigate the usefulness of our wide-angle ultrasound diagnostic device, there is a need to determine whether similar results can be obtained in older adults who have difficulty during the examination, as well as people with low vision in both eyes.

In the future, we expect that wide-angle B-mode echography, which enables simple and noninvasive imaging, will aid in the determination of ocular morphology in various diseases, such as retinal detachment, choroidal detachment, equatorial degeneration, and macular disease; it will be useful for research related to such diseases, as well as detailed characterization of ocular morphology.

## Figures and Tables

**Table1:** AL, HD, VD, HD/AL, VD/AL, and HD/VD obtained by wide-angle ultrasound diagnostic echography and MRI

	WAB (mm)	MRI (mm）	Difference 95% CI	β	R^2^	*p*-value
AL	25.22±1.47 [24.57, 25.88]	25.24±1.46 [24.59, 25.89]	–0.32 to 0.01	0.995 [0.976, 1.013]	1.00	<0.01
HD	22.33±0.84 [21.95, 22.70]	22.55±0.90 [22.10, 22.90]	–0.32 to 0.1	0.902 [0.750, 1.179]	0.81	<0.01
VD	22.77±0.91 [22.37, 23.18]	22.88±0.92 [22.47, 23.29]	0.00–0.21	0.966 [0.853, 1.097]	0.93	<0.01
HD/AL	0.89±0.03	0.89±0.03	0.00–0.01	0.899 [0.642, 1.020]	0.80	<0.01
VD/AL	0.91±0.05	0.91±0.05	0.00–0.01	0.975 [0.867, 1.070]	0.95	<0.01
HD/VD	0.98±0.04	0.98±0.04	0.00–0.01	0.917 [0.763, 1.152]	0.83	<0.01

All parameters demonstrated significant correlations for wide-angle ultrasound diagnostic echography and MRI. WAB and MRI measurements are shown as mean±standard deviation [95% CI]. AL, axial length; HD, horizontal distance; VD, vertical distance; WAB, wide-angle B mode; MRI, magnetic resonance imaging; CI, confidence interval; β, regression coefficient

## Data Availability Statement

The datasets used and/or analyzed during the current study are available from the corresponding author on reasonable request.
